# Scrambler Therapy for Chronic Neuropathic Pain: Why the Evidence Base Has Not Converged, and What Standardization Would Require

**DOI:** 10.3390/jcm15197499

**Published:** 2026-09-26

**Authors:** Hyungin Cho, Heeyeon Yoon, JinHan Lee, Junhwan Jung, Ingyu Lee

**Affiliations:** 1 Eco Samsung Orthopedic Clinic, 25 Sebyeongnam-ro, Jeonju-si 54819, Jeollabuk-do, Republic of Korea; 2 Korean Society of Scrambler Therapy Medicine (KSTS), Room 202, 33 Hanaunan 1-gil, Naun-dong, Gunsan-si 54130, Jeonbuk-do, Republic of Korea

**Keywords:** scrambler therapy, neuropathic pain, neuromodulation, electroanalgesia, research standardization, sham control

## Abstract

Scrambler therapy (ST) is a non-invasive transcutaneous electroanalgesic technique proposed to deliver synthetic “non-pain” information rather than conventional gate-control stimulation. Despite two decades of favorable clinical signals and few reported serious harms, guideline endorsement remains limited and quantitative syntheses diverge. This narrative review examines why the ST evidence base has not converged and what standardization would require. We identify six recurrent structural problems: an unresolved sham-control paradox, operator dependence, device heterogeneity, an unresolved medication interaction, outcome timing, and post hoc attribution of negative results to protocol deviation. A Korean national health technology reassessment independently reproduced comparator-dependent effect estimates and very low certainty. Public Health Insurance Review and Assessment Service (HIRA) price disclosure records further show that institutions reporting the relevant administrative item increased from 189 in 2021 to 871 in 2026, while the public record cannot identify the device used. As preliminary proposals rather than validated standards, we offer a 22-item reporting set, a conceptual phenotype-stratified dismantling trial, and a prospective registry model. We argue that intervention definition, delivery fidelity, and trial architecture limit the interpretability of the evidence and should be standardized before additional large confirmatory trials; standardization would make results interpretable, not predetermine their direction.

## 1. Introduction

Chronic neuropathic pain is managed with drugs of modest average effect, high numbers-needed-to-treat, and dose-limiting harms. This gap sustains interest in neuromodulation, from spinal cord stimulation at the invasive end to transcutaneous techniques at the other.

Scrambler therapy (ST) sits at the non-invasive end but claims a categorically different mechanism. First described in patients with intractable pain from abdominal malignancy [1] and subsequently formalized as a device-based modality [2], ST delivers low-amplitude stimulation through surface electrodes sited in pain-free skin bracketing the painful territory, assembling synthetic action-potential waveforms into continuously varying sequences under algorithmic control. The stated intent is not to close a spinal gate by recruiting large-diameter afferents, but to occupy C-fiber surface receptors with information the central nervous system parses as “non-pain”, forcing re-encoding of the pain percept over repeated sessions [3].

This distinction generates different predictions about electrode placement, dose response, responder selection, and what constitutes a valid control, as well as a distinctive failure mode: delivered as though it were transcutaneous electrical nerve stimulation (TENS), ST is, on its own theory, not being delivered at all.

Two decades on, the field presents a striking discordance. Trials and pooled syntheses repeatedly report reductions exceeding conventional thresholds for clinical meaningfulness, persisting weeks to months after a ten-session course [4,5], and a dedicated systematic review of harms reported a low incidence of adverse events and no serious device-attributable events, although ascertainment was largely passive [6]. However, the American Society of Clinical Oncology places ST among approaches for which “proof of benefit is lacking” while conceding it is reasonably safe and reasonable to consider [7], and a 2023 review in a general medical journal reached a similarly hedged position [8].

The usual explanation is that the trials are small. We argue this is incomplete and, as a research prescription, misleading. They are small, but they are also mutually incommensurable—differing in device, electrode siting, titration, operator training, concomitant sodium-channel-blocking medication, and follow-up horizon. Pooling incommensurable trials in greater numbers does not produce convergence; it widens the confidence interval around an uninterpretable average.

This review restates ST’s mechanistic premises, appraises the clinical evidence, sets out six structural problems responsible for the failure to converge, proposes a preliminary reporting set and a conceptual design template, and examines the Korean administrative and regulatory record.

## 2. Review Scope and Approach

This is a narrative review. It is not a systematic review, was not registered, and makes no claim to exhaustive retrieval. We state this explicitly because the argument below concerns methodological transparency, and a review advancing that argument should be held to it.

We began from the four existing evidence syntheses [5,9,10,11] and the two general reviews framing current clinical understanding [8,12], working outward through reference lists and forward through citing articles, adding primary trials, guideline statements, editorial correspondence, one retraction notice, and one national health technology assessment report identified through the Korean regulatory record [13]. Correspondence was included deliberately: several of the field’s substantive methodological disputes have been conducted there rather than in primary reports [14,15,16].

Where an assertion about clinical effect is quantitative, we take it from the synthesis that generated it and attribute it there rather than recomputing. Where syntheses disagree, we report the disagreement rather than adjudicating it. Separately, to characterize the Korean administrative record, we queried the HIRA non-covered treatment cost disclosure system by administrative region on 18 August 2026 and summed institutions reporting a price under the relevant administrative item. The source and query procedure are described in the section on the Korean case. No patient-level data were accessed. Generative AI assistance was limited to language editing, structural refinement, citation renumbering, reference-formatting support, manuscript formatting, and conceptual figure design; all source selection, evidence characterization, quantitative statements, and HIRA aggregation were reviewed and verified by the authors.

Depth of source examination: Sixteen sources available in full text without restriction were examined completely, including methods and funding statements; this is how the device and funding characteristics reported below were identified, and they are not recoverable from abstracts. For the remainder, principally subscription-access articles are of secondary importance to our argument; our characterization rests on abstracts, publisher records, and descriptions in the syntheses that included them.

Limitations: Our selection inherits the citation biases of the syntheses we started from, and we did not extract individual patient data or independently verify the analyses we cite. The HIRA snapshot is descriptive and depends on region-by-region public queries; the system provides no device field, patient denominator, or national distribution summary, so it cannot establish device-specific uptake, utilization, or treatment quality. It also concerns a single health system; regulatory and reimbursement arrangements differ elsewhere, and the Korean observations should not be generalized without comparable data from other jurisdictions.

Author relationships: Two of the authors hold office in a society that trains practitioners in scrambler therapy, and one delivers it in clinical practice (see Conflicts of Interest). These relationships could influence which sources we weighted, how we interpreted negative trials, and the reporting framework we propose, which would, if adopted, formalize practices our society teaches. We have tried to guard against this by attributing quantitative claims to the syntheses that generated them, by reporting disagreement rather than resolving it, and by applying the same full-text scrutiny to the most favorable trial in the literature [4] that we applied to the least. Readers should nonetheless weigh our interpretations accordingly, and the reporting set proposed below requires independent external evaluation before it could be treated as a standard.

## 3. Mechanistic Premises

### 3.1. What ST Is Not

TENS is grounded in gate control theory: high-frequency, low-intensity stimulation recruits Aβ afferents that engage dorsal horn inhibition. It is typically self-administered, uses fixed or manually adjusted parameters, places electrodes over the painful region, and produces analgesia largely coextensive with stimulation, with tolerance over repeated use [8].

ST diverges on every dimension. The published parameters are specific: sixteen synthesized action-potential types assembled into information packets of 43–52 Hz and 0.7–10 s, each generated by an algorithm that accounts for prior outputs and varies potential type, frequency, duration, and modulation amplitude. Output is restricted to 256 validated strings, and all parameters other than intensity are automated and not modifiable by the operator [3]. The maximum current is 3.5–5.5 mA, and because frequency never exceeds 52 Hz, the mean energy per second is generally lower than conventional TENS [4]. Electrodes are placed in pain-free skin bracketing the painful territory, explicitly not over it. Intensity is titrated by a trained operator to a defined perceptual endpoint. The effect is claimed to accumulate across roughly ten consecutive daily sessions and to persist afterwards.

The persistence claim is mechanistically load-bearing: gating predicts stimulus-locked analgesia, while the proposed rewriting of an aberrant pain representation predicts progressive carry-over benefit and therefore an appropriate primary endpoint at or after the end of the course. Figure 1 summarizes the conceptual contrast in electrode topology, waveform structure, and predicted temporal profile.

### 3.2. The “Active Principle of Information” Hypothesis

Marineo’s formulation holds that chronic neuropathic pain is sustained by a maladaptive, self-reinforcing central representation—an information problem as much as a transmission problem [3]. The analgesic target is the content of the afferent signal rather than its magnitude.

Observations compatible with the hypothesis. Reductions outlasting stimulation appear in multiple independent cohorts [4,5,11,17]. Central changes are measurable: a sham-controlled trial in chronic post-burn neuropathic pain found greater pain reduction than sham at two weeks (median visual analog scale 6 → 3 versus 7 → 6; between-group *p* < 0.001), alongside decreased cerebral blood volume in bilateral orbitofrontal and superior frontal gyri and increased cerebral blood volume in the pre- and postcentral gyri of the burned limb’s hemisphere [18]. Response varies by phenotype: in 27 patients with drug-resistant neuropathic pain, cluster analysis of Neuropathic Pain Symptom Inventory [19] profiles found differential response across three clusters (time × cluster *p* = 0.003), favoring paroxysmal-over persistent-predominant pain. The investigators proposed that responders retain relatively preserved C-fibers [20].

Three features of these two studies qualify their use and are only visible in the full text. In the burns trial, the sham group also improved significantly from baseline (*p* = 0.001); six of twenty patients allocated to active treatment did not return for post-treatment imaging because their pain had improved, so the analyzed sample was 14 versus 23 rather than the randomized 43; and although the trial was marked as double-blinded, its methods state that the therapist was aware of the allocation schedule, while participants and evaluators were blinded [18]. In the phenotype trial, the device was a GEOMC NPC3 rather than an MC-5A, and the funder was the device manufacturer, although the authors report that the funder had no role in study design, analysis, or reporting [20].

None of these observations directly demonstrate the proposed mechanism. Sustained analgesia is compatible with the information hypothesis but also with nonspecific treatment effects, expectancy, natural history, and regression to the mean; changes in cerebral blood volume indicate altered activity in pain-related networks without identifying the process that produced them. We therefore treat “information rewriting” throughout as a mechanistic hypothesis whose predictions are testable—principally by the dismantling design set out below—rather than as an established biological process.

Unresolved issues include whether the specific waveform library and sequence algorithm are necessary, or whether any sufficiently non-repeating C-fiber-range stimulation delivered outside the painful field would suffice. This is the field’s most consequential open question and has never been tested using a dismantling design. It is also unresolved whether receptor capture is literally correct at the level of primary afferent physiology, and whether anticonvulsants truly attenuate response.

## 4. Clinical Evidence

Table 1 summarizes the principal controlled and prospective studies.

Cancer pain: ST entered the literature here, with dramatic reductions reported in refractory pain [1,2]. Open-label work in mixed cancer populations reported consistent benefit [22,35,36], a 219-patient single-center series reported durable reductions [24], and a Korean case series contributed early independent replication [37]. An open-label randomized trial from a single Indian center allocated 80 patients with head, neck, and thoracic cancer pain to WHO-ladder pharmacotherapy with or without daily 40 min ST, reporting greater pain reduction and lower morphine requirements [32], with a companion report of the same cohort adding improvement across all WHOQOL-BREF domains [33]. The relationship is transparently declared, but the two reports are easy to mistake for two trials: a reader assembling an evidence table from titles and abstracts would count 160 patients where there are 80. The quantitative syntheses appear to have avoided this: the dose–response analysis specifies five placebo- or sham-controlled and three routine-care-controlled trials [9], a split reconcilable with the published randomized literature only if this cohort is counted once. A systematic review restricted to cancer pain concluded that the evidence is insufficient in quality to establish efficacy [10].

Chemotherapy-induced peripheral neuropathy: CIPN is the most studied indication and the most instructive, because the highest-quality equivocal data sit here. Early pilot work reported substantial improvement [21], as did open trials [22,38]. A randomized phase II trial of 50 patients found that 56% of ST recipients versus 28% of TENS recipients achieved at least a 50% pain reduction [25]; its crossover phase showed the same direction [31]. Against the sham, the picture differs: a pilot sham-controlled trial in 35 patients found no difference at day 10, 28, 60, or 90 for average pain, the Brief Pain Inventory, or EORTC CIPN-20 [26]. A recent pilot reported improvements sustained at six months [34], and a systematic review of neuromodulation for CIPN reached conclusions consistent with the guidelines [39]. Pediatric data are preliminary [40,41].

The ASCO guidelines reflect this precisely: duloxetine is the only agent with adequate supporting evidence for established painful CIPN, while ST, acupuncture, and exercise are suggestive but unproven [7]. The CIPN literature is the clearest demonstration that the field’s problem is design rather than direction: uncontrolled studies are uniformly strongly positive, while controlled studies—heterogeneous in device, placement, titration, and sham construction—are correspondingly heterogeneous in result.

Non-cancer peripheral neuropathic pain: Korean investigators provided the first dedicated reports on postherpetic neuralgia refractory to conventional treatment [42]. The most frequently cited controlled evidence remains a trial of 52 patients with postsurgical neuropathic pain, postherpetic neuralgia, or spinal canal stenosis, in which the mean visual analog scale fell from 8.0 to 0.78 at one month versus 8.11 to 5.84 with revised pharmacotherapy, with persistence at three months and elimination of opioids in 11 of 17 cases [4]. Four features are recoverable only from the full text. Patients were assigned consecutively in enrollment order, alternating within pain stratum, and the report states that allocation was not concealed. The two research assistants who delivered ST also collected pain assessments in both arms. The control arm had its failed regimen switched while the ST arm continued with its failed regimen unchanged, so the contrast is ST plus ineffective drugs versus revised drugs. In addition, patients using antiseizure medications were excluded, as were TENS responders—the consequences of the former are developed below. None of this establishes that the effect is spurious; it establishes that the largest effect size in the literature comes from the trial with the least protection against selection and ascertainment bias.

For lower back and mixed traumatic neuropathic pain, a sham-controlled study of 30 patients reported reduced pain with differential gene expression [23], while a larger double-blind sham-controlled trial in 47 military service members found no between-group difference in pain, analgesic use, or quality of life at any time point, with pain falling in both arms (4.3 → 2.5 for active; 4.8 → 3.2 for sham) and converging at one month [27]. Baseline pain averaged 4.5 of 10 versus 8.0 in the trial reporting the largest effect; whether ST’s effect concentrates in severe refractory pain is testable, but is not, in the absence of such a test, an explanation. More troubling is the fact that mixed linear modeling of all 36 collected scores returned a significant group × time interaction (*p* = 0.023), while the same modeling of the three scheduled assessments returned none (*p* = 0.52).

For painful diabetic polyneuropathy, we identified no controlled trials. A single-arm prospective pilot in nine patients delivered ten 45-min sessions and measured outcomes at six months: pain improved significantly at the eighth to tenth sessions and at one month, while every other outcome at every other time point was not significantly different from baseline. The authors conclude that ST may have short-term efficacy but limited long-term effects [29]. A narrative review of neuromodulation options for this indication concurs [43]. Given its prevalence, the absence of controlled evidence is the largest gap in the clinical literature. A study on failed back surgery syndrome was retracted and is not considered evidence here; see below [44]. For complex regional pain syndrome, only case material exists [12,45].

Central neuropathic pain: A trial randomized 22 patients with neuromyelitis optica spectrum disorder to ST or sham over ten weekdays. Primary outcomes were acceptability and feasibility; efficacy was secondary. The median numeric rating scale fell from 5.0 to 1.5 with active treatment, while the sham showed no significant decrease, with improvements in anxiety and depression sustained at 30 days but not at 60 days [17]. The design is unusually clean: a stable severe pain phenotype, a single trained technician delivering all interventions, and the device kept behind a curtain and powered on in both arms so that the end-of-session alert sounded identically in each. The sham nonetheless illustrates rather than escapes the paradox below: sham patients received no stimulation at all, and a patient who feels nothing for ten sessions has information about allocation, so the trial was single-blind. The 60-day result also sits against the synthesis, reporting that effects peaked near day 40 and persisted at day 90 [9,46]. A second sham-controlled trial has since been reported. Twenty patients with contralesional post-stroke pain were randomized to ST or sham as an adjunct to stable medication, receiving five consecutive daily 40 min sessions, with both patients and assessors blinded. Mean pain change favored ST both immediately (−3.73 versus −0.94) and at four weeks (−2.57 versus −0.25; *p* = 0.012 and 0.004), but the proportion achieving more than a 50% reduction fell from 70% versus 10% immediately (*p* = 0.006) to 30% versus 10% at four weeks, becoming non-significant [28]. The course was five sessions instead of ten. The investigators have begun a larger trial comparing five sessions against ten—the first dose-finding study in this literature—and precisely the work that this review argues is overdue.

This subsection rests on 42 patients across two small trials; we include it because the mechanistic implication is genuine, not because the evidence supports clinical recommendation.

## 5. Safety

A PROSPERO-registered systematic review searching six databases through to December 2021 identified 36 studies comprising 1152 patients. Three patients experienced an adverse event: two cases of contact dermatitis and one case of minor ecchymosis, all resolving without intervention. The composite complication rate was 0.26%, with zero serious adverse events [6]. Against the 30–40% complication rate of spinal cord stimulation cited in the same review, this is a difference in reported rates of orders of magnitude, subject to the ascertainment qualification below. The relevant counterfactual is not no treatment but gabapentinoid sedation and falls, duloxetine adverse effects, or opioid exposure.

Two qualifications keep this from being a stronger claim than the data support. First, the review’s own extraction table records the surveillance method per study, and for the majority, it is unstated or passive self-report; only one study applied a standardized toxicity instrument. The rate of 0.26% is derived predominantly from passive surveillance and is a lower bound on reported harm, not an estimate of occurring harm, and the same equal-ascertainment deficiency that limits the efficacy literature limits the safety literature. Second, the review states that no included study reported financial support from the manufacturer; however, one included study declares that its funder was the manufacturer of the device used [20]. Device and sponsorship attributes pass unnoticed when they are not required reporting items.

Standard contraindications apply and should be stated in any protocol: implanted electrical devices, carotid sinus placement, broken or infected skin, pregnancy, and application over a known malignancy without oncological consultation.

## 6. What the Syntheses Establish

Four syntheses bear on efficacy. A systematic review conducted according to the Preferred Reporting Items for Systematic Reviews and Meta-Analyses (PRISMA) statement, with dual independent extraction, surveying studies from 1950 to October 2021, concluded that ST is safe, with the potential for significant analgesic benefit in neuropathic pain, most robustly in CIPN [5]. A systematic review restricted to cancer pain found the evidence to be insufficient [10]. A meta-analysis of seven randomized trials (287 patients) reported that ST marginally decreased pain scores, with a standardized mean difference (SMD) of −0.85 (95% CI −1.66 to −0.03) and I^2^ = 89.5%; analgesic consumption fell significantly (SMD −0.54, 95% CI −0.93 to −0.14, I^2^ = 0%, two trials). Critically, no significant efficacy survived stratification by methodological quality, by disease, or by follow-up period [11]. A pairwise and dose–response meta-analysis of eight trials (350 participants) reported a clinically important short-term reduction, given as −2.16 (95% CI −2.85 to −1.47) in one section of the text and approximately −3 points in the abstract summary, most likely reflecting the two pooling models reported, with pain continuing to fall after the course ended. The largest difference from baseline was near day 40, and scores below baseline were observed until day 90 [9].

A fifth synthesis sits outside the journal literature and is, to our knowledge, not cited anywhere. In 2021, Korea’s National Evidence-based Healthcare Collaborating Agency (NECA) conducted a statutory reassessment, systematically reviewing 30 studies with duplicate screening, extraction, and risk-of-bias appraisal [13]. Its pooled estimates locate the disagreement precisely in the choice of comparator. Against the sham, the pooled SMD was −1.17 (95% CI −2.81 to 0.47; I^2^ = 93%), and excluding the single trial whose sham electrodes were sited at the most painful area, it was −0.08 (95% CI −0.49 to 0.33; I^2^ = 0%). Against TENS, ST was superior: SMD −0.90 (95% CI −1.35 to −0.44; I^2^ = 12%), with a pooled odds ratio of 3.40 (95% CI 1.24 to 9.30) for at least 50% pain reduction. In combination with drug therapy, it outperformed drug therapy alone (SMD −2.62, 95% CI −4.42 to −0.82; I^2^ = 93%). Blinding of both participants and the outcome assessment was rated high risk in 40–50% of the randomized trials, funnel plots were asymmetric, and Grading of Recommendations Assessment, Development and Evaluation (GRADE) certainty was very low for every outcome. The committee nonetheless judged the technique safe and clinically useful and issued a conditional recommendation, specifying that blinded comparative studies, long-term outcome data, and cost-effectiveness analysis were required [13].

What matters here is provenance and shape. An arm’s-length national agency, working with a substantially broader evidence base than the randomized-trial meta-analyses cited above and with no primary-trial involvement, arrived at exactly the configuration this review takes as its problem: a consistent direction of effect, a null against sham, heterogeneity it could not resolve, certainty at the floor of the GRADE scale, and endorsement issued conditionally rather than granted or withheld. The fact that the non-convergence survives a change of appraiser, jurisdiction, and evidence base indicates that it is a property of the primary literature rather than of any one reviewer’s method. The remedy the agency named was blinded comparative studies; our argument is that the blinding deficiency it identified is the structural paradox set out below, which no quantity of further trial activity resolves until the sham itself is redesigned.

One structural feature deserves comment, as an observation about a small field rather than an allegation. The systematic review, the safety review, and the general medical review share authorship with each other and with a substantial proportion of the primary trials they assess [4,5,6,8,17,21,22,25,26,31,38,47]. This is unremarkable where few investigators are active, and those reviews declare their interests, but it does mean that genuinely independent synthesis is scarce, and it is the syntheses produced outside that group [9,11] that report the more equivocal estimates. We do not claim that the pattern is causal, only that it is a reason to weight independent syntheses at least as heavily and to commission one from investigators with no primary-trial involvement.

Four observations follow.

First, when two competent syntheses of the same eight or fewer small trials reach conclusions supporting opposite clinical recommendations, the honest reading is that the underlying data do not determine an answer. The divergence turns on the pooling model, on whether heterogeneity is a signal to explain or a quantity to absorb, and on how methodological quality enters the estimate.

Second, the dose–response finding is the most mechanistically informative result in the quantitative literature: post-treatment continuation and a later peak are difficult to reconcile with gating and are consistent with, though not specific to, the proposed information-rewriting hypothesis.

Third, the fact that “equal ascertainment” is the most deficient standard on the MASTER methodological appraisal scale is a precise technical statement of the sham problem, not a finding about sample size. The small-study analysis returned a Luis Furuya-Kanamori index of 3.49—a value indicating major asymmetry, which the authors present as evidence that smaller studies underestimated the effect. This reading is possible. An index of that magnitude indicates marked small-study asymmetry, which may arise from publication or reporting bias but also from between-study heterogeneity, differences in methodological quality, or chance; asymmetry alone establishes neither the presence of publication bias nor its direction, and the authors label the analysis as depicting only the possibility of publication bias [9]. Given that the largest single effect comes from an unconcealed, alternately allocated trial with provider-assessed outcomes, we regard the asymmetry as unresolved rather than favorable.

Fourth, a difference that is invisible at the level of abstracts: contributing trials differ in session length by a factor of two—from 30 min [20,25,26,38] to 60 in the first CIPN pilot [21]—and in channel count, electrode siting rule, and intensity ceiling. All are described simply as “ten sessions of scrambler therapy”.

## 7. Six Structural Problems

### 7.1. The Sham Paradox

ST in therapeutic settings produces unmistakable paresthesia. A perceptually indistinguishable sham must therefore deliver comparable stimulation, which may not be inert; a sub-perceptual sham is inert but unblinding. Marineo has argued that an “ineffective perception threshold” does not exist for this modality and that sham electrodes must be sited well away from the painful field [16]. This is not a late defensive move: the 2012 trial’s own discussion states that blinded studies have been hard to devise because placement and dose must be titrated to pain relief before treatment begins, offering, in place of a sham, the argument that a 91% reduction exceeds any plausible placebo magnitude [4]. This argues that the sham problem should be regarded as unsolved, not dispensable.

The paradox is visible in practice. The post-burn imaging trial used sham stimulation at a non-therapeutic level, with electrodes at the same site in both arms. The operator knew the allocation by necessity; the report is nonetheless titled as double-blinded [18]. We raise this not to fault those investigators, whose methods state the arrangement plainly, but because the field’s own device literature concedes that ST admits only partial double-blinding—a constraint not carried into the titles and abstracts that downstream syntheses inherit.

A second observation is sharper and concerns what “sham” means. In the sham-controlled CIPN trial, the active arm received sessions on the dermatomes above the painful areas, while the sham arm received treatment on the back, typically at L3–5, where the nerve roots serving the painful territory enter the spinal cord [26]. This is not an inert control: it is stimulation using the same device at a site connected to the same segmental level. Based on ST’s own account of dermatomal transmission, it is a plausible active site. The trial’s authors list that the sham may not have been a sham among the possible explanations. If so, the trial may have compared two applications of ST rather than ST versus an inert control. We cannot establish that it did: no physiological or clinical evidence shows that stimulation at that site is therapeutically active, and we raise the possibility as a limit on interpretation, not as an explanation of the null result. What can be said is that classifying this trial as sham-controlled [9,11] rests on an assumption of inertness that its design does not verify.

Four distinct problems are conflated under the heading of the sham paradox, and they have different consequences: (i) inadequate blinding, in which participants or assessors can infer allocation, permitting expectancy and ascertainment bias that tends to favor the active arm in practice; (ii) a potentially biologically active sham, which would bias estimates toward the null; (iii) a biologically inert but perceptually distinguishable sham, which provides a valid contrast in principle but is exposed to (i); and (iv) uncertainty surrounding inertness, the most common situation in this literature, in which the direction of any bias cannot be stated. A negative sham-controlled trial should therefore be read as a genuine null unless there is specific evidence that its sham was active.

Before proposing solutions, we record one already attempted and largely lost. In 2013, Campbell and colleagues reported a randomized, double-blind trial of ST against a purpose-built active sham delivering a barely perceptible but non-therapeutic sensation—precisely the construction that the paradox calls for. Fourteen patients with painful CIPN received ten 50 min sessions; there was no significant difference in the primary endpoint [48]. The trial is small and settles nothing on its own, and because it is available only as a conference abstract, its methods and results cannot be appraised to the standard of a full peer-reviewed report; we treat it as bibliographic rather than confirmatory evidence. What matters here is its bibliographic fate. The study that comes most closely to resolving the field’s central design problem is the least accessible and least cited, and is absent from the syntheses that define current effect-size expectations; its absence from these limits any claim that the key design question is unanswerable. Figure 2 summarizes the sham-control dilemma and the active-comparator and dismantling approaches that can address it.

There are three routes forward:

1. Active-comparator designs: comparison against TENS in a therapeutic setting, or a standardized pharmacological regimen, preserves expectancy while answering the comparative-effectiveness question clinicians and payers require.
2. Dismantling designs: Compare full-protocol ST against ST with one mechanistically critical element removed—algorithmically varying sequences replaced by a fixed repeating waveform at identical perceptual intensity, or electrodes placed within rather than outside the painful field. Both arms are perceptually indistinguishable; the contrast isolates the proposed active principle. This has never been applied to ST and is arguably the highest-priority study the field could run.
3. Blinding-integrity and perceived-benefit measurement as reported outcomes: One trial has come close, and its result is the most instructive datum in the sham literature. In 47 military service members, the sham was delivered at sub-threshold intensity with leads outside the affected dermatomes—inert based on ST’s own theory, and therefore not vulnerable to the objection that the sham stimulated the painful field. Before learning their allocation, 90% of the blinded sample rated the treatment a partial (63%) or complete (27%) success, 94% would use it again, and acceptability did not differ between arms (42.7 versus 41.1, *p* = 0.345) [27]. This is strong evidence that blinding held, and equally strong evidence that perceived benefit here is substantially generated by something other than the specific intervention. A field seeking specific efficacy cannot cite patient satisfaction as support.

### 7.2. Operator Dependence Without a Competency Standard

ST delivery involves judgment at several points: identifying the territory, selecting channel topology, siting electrodes in pain-free skin, titrating to a defined endpoint with repositioning when it is not achieved, and managing the residual painful area.

The evidence is direct. In a multicenter analysis of 147 patients at three centers, overall success—at least 50% relief persisting beyond one month—was 38.1%, and treatment site independently predicted success in multivariate logistic regression, with odds ratios of 6.87 (95% CI 1.60–29.51) and 12.29 (95% CI 1.73–87.43) [30]. After adjustment for pain type, etiology, baseline severity, compliance, psychopathology, and concomitant medication, where a patient was treated multiplied the odds of success several-fold. Those intervals are wide, and site location is a composite of every difference between institutions. Operator technique is therefore a plausible contributor to a site effect of that size, although it cannot be separated from other center-level factors in these data.

The one study designed to look for this reinforces it. A multicenter retrospective analysis of 201 patients at nine Italian centers states that its primary aim is the analysis of efficacy in relation to differing usage conditions and learning curves [49]. Aggregate results were strong (mean numeric rating scale: 7.41 → 1.60), but when disaggregated by center, the proportion achieving at least 50% relief was 50.0%, 72.4%, 75.0%, 80.0%, 81.8%, 84.2%, 87.7%, 92.3%, and 97.8% [3]. Center sizes were unequal (*n* = 4 to 65), but the center reporting 50% treated ten patients, and the one reporting 97.8% treated forty-five. The developer adds that within a single team, more experienced practitioners achieved complete intra-session pain resolution where less-experienced operators had failed [3].

Investigators concur, accounting for a 22% mean reduction versus the 30–90% reported elsewhere. The phenotype trial’s authors listed operator inexperience among three candidate explanations, despite a pre-trained operator following a written protocol under supervision [20].

One trial has addressed this by design: the NMOSD investigators trained a single technician to perform every intervention, because effect has been shown to vary across technicians [17]. This does more for the interpretability of a 22-patient trial than doubling its sample would have.

Beyond this, no published trial was identified that reports operator training in a form permitting replication—hours, supervised cases, competency assessment, or inter-operator agreement on placement—so between-trial heterogeneity in effects cannot be distinguished from between-operator heterogeneity in delivery. If a two-fold range in response rate tracks operator experience, that is not a nuisance variable for a limitations paragraph; it is an effect modifier exceeding the treatment effects most trials are powered to detect.

### 7.3. Device Heterogeneity and Label Drift

Scrambler Therapy^®^ is a proprietary technology with a specific referent. This is a scientific matter as much as a trademark issue, because the waveform library and sequence algorithm are the proposed active principle.

Consider three trials examined in the full texts. One describes an MC-5A Pain Scrambler from Competitive Technologies, Fairfield, Connecticut [18]. A second describes a GEOMC Pain Scrambler (model MC-5A) from Seoul [17]. A third describes a GEOMC NPC3 device from Seoul [20]. A reader cannot determine from the reports whether these involve one device, two, or three: the first two share a model designation but name different companies on different continents, and the third shares a company with the second but not a model. The likely explanation—manufacturer versus distributor, differing model lines—is stated in none of the three, and a synthesis pooling them asserts an equivalence that none of its sources establishes.

The clearest illustration is a retraction. A study reported under the name “Scrambler Therapy” in failed back surgery syndrome was withdrawn after publication; the retraction notice states that the trademark may be used only for approved devices, which do not include the Pain Jammer ENS-1140 used in that study, and that the authors do not agree with the retraction [44]. We cite the notice rather than the retracted report and draw no clinical inference from the study. The episode is instructive for a different reason: the non-equivalence of the intervention was established after publication, by a trademark holder, rather than before it, by peer reviewers or meta-analysts comparing the reported procedure against the published protocol [3]. Where the identity of an intervention depends on procedural fidelity, that comparison is a reviewer’s job, and a trademark holder should not be the mechanism of last resort.

The developer’s own standard is stricter than the field’s practice: he describes the technology as residing in an original equipment manufacturer (OEM) module that a manufacturer cannot alter without invalidating clinical characteristics, and equates any modification of the emissions in form and temporal organization to modifying a drug’s chemical formula [3]. On that standard, establishing that two devices are the same intervention requires evidence about emission structure rather than frequency, pulse width, and intensity—evidence never published for any alternative device, and demanded by no journal, reviewer, or reimbursement system.

Every ST publication should state the device manufacturer, model, and clearance identifier; systematic reviews should pre-specify device eligibility and conduct device-stratified sensitivity analyses; and registries should record device identity at the encounter level.

Protocol-concordant ST: For interpretation, we propose that an intervention be regarded as protocol-concordant scrambler therapy when four criteria are met: (i) the device is identified by manufacturer, model, and regulatory clearance as a Scrambler Therapy^®^-designated device incorporating the developer’s emission library; (ii) electrodes are placed in pain-free skin bracketing the painful territory; (iii) intensity is titrated by a trained operator to the protocol’s intra-session endpoint; and (iv) the course approximates the specified ten-session dose, with the number, schedule, and inter-session interval reported (item 9 of the Scrambler Therapy Minimum Reporting Set (ST-MRS); Table 2) [3]. The developer’s protocol specifies consecutive daily sessions; published courses already vary [28], and whether spacing sessions over several weeks modifies the effect has not been tested. Studies failing (i) evaluate a broader category of patterned transcutaneous electroanalgesia; studies meeting (i) but not (ii)–(iv) evaluate the device outside its protocol. This review evaluates the literature reported as scrambler therapy and treats concordance with these criteria as a characteristic to be reported, not as an inclusion criterion, which is why several of the trials discussed above fall short of one or more criteria. The criteria are ours, derived from the published protocol, and are offered for testing rather than as a definition that the field has adopted.

### 7.4. Concomitant Sodium-Channel-Blocking Medication

Proponents assert that concomitant anticonvulsants attenuate responses by interfering with action potential propagation, and invoke this claim to explain equivocal results. It is not informal: the developer states that anticonvulsants at a high dose may inhibit effectiveness, that patients should be weaned before or during initial treatment, and that these warnings are contained in device documentation lodged in FDA and CE authorization procedures [3]. A contraindication carried in a regulatory file has a different status from an observation in a discussion section, and is, so far as we can determine, supported by no prospective clinical test.

Three observations bear on its evidentiary status. First, and we believe this has not previously been stated: the foundational controlled trial—the source of the 91% figure against which later results are judged disappointing—excluded patients using antiseizure medications altogether [4]. The confound runs in one direction: the study reporting the largest effect enrolled nobody from the group in which the effect is said to be attenuated, while studies reporting modest effects enrolled populations in which anticonvulsant use approached universality [20]. Whether the medication explains that difference or merely tracks it cannot be recovered from designs constructed this way; the appeal to sodium-channel blockade draws its plausibility from precisely the confound it is offered to explain.

Second, the multicenter predictive analysis explicitly evaluated membrane-stabilizer use among its candidate covariates and did not find it predictive; the only medication variable approaching significance was antidepressant use, in the direction of failure (OR 0.47, 95% CI 0.18–1.02, *p* = 0.056) [30]. A hypothesis invoked for two decades has been tested once, incidentally, and not supported.

Third, in the phenotype trial, anticonvulsant use was entered as a fixed effect in the mixed model, so the reported treatment effect is already adjusted for it; however, the authors offer anticonvulsant use as an explanation for the modest effect size in their discussion [20]. The claim is being invoked interpretively in studies whose own analyses controlled for it.

The remedy is pre-specified stratification on baseline sodium-channel-blocker use, protocolized tapering arms where permissible, or mandatory reporting of medication class, dose, and stability as a minimum. Until then, the interaction is best regarded as an unresolved effect-modification hypothesis: the available evidence neither confirms nor excludes it, and it should be tested prospectively rather than invoked interpretively in either direction.

### 7.5. Outcome and Follow-Up Architecture

If the effect continues to develop after the course and peaks around day 40 [9], a trial whose primary endpoint is pain on the final treatment day measures the wrong thing at the wrong time. As a hypothesis-driven proposal for future trials rather than an empirically established window, we suggest a primary endpoint at day 28–42, with the end-of-course measurement retained as a key secondary endpoint and follow-up to at least 90 days, so that the trajectory itself, including any early decline, is characterized rather than assumed. Four observations bear on this and do not agree: non-persistence at 60 days in the NMOSD trial [17]; dissipation of individual responses by day 30 in the sham-controlled CIPN trial [26]; loss of the responder advantage by four weeks in the post-stroke trial [28]; and, in the diabetic polyneuropathy pilot, significance at one month with nothing significant thereafter [29]. Set against a synthesis reporting scores below baseline through to day 90 [9], this is an empirical question that the field has not settled, rather than a parameter that it may assume. The inconsistency cuts both ways: where effects decline by 30–60 days, a later endpoint would record a smaller effect than an end-of-course endpoint, and that is a result the field needs rather than one to design around.

Endpoint selection requires attention beyond pain intensity: in the phenotype cohort, Brief Pain Inventory interference improved significantly, while the pain composite did not [20]. Trials should include function and interference, sleep, analgesic consumption with a defined tapering protocol, and responder analyses at ≥30% and ≥50% alongside mean differences.

### 7.6. The Protocol-Deviation Defense

The sixth problem is epistemological and follows from the first five. Because ST is operator-, device-, and medication-sensitive, any negative result can be attributed to incorrect delivery. The developer’s review attributes the double-blind, sham-controlled lower-back-pain trial’s outcome to sham electrodes placed too close to the painful area and to double-blinding preventing normal operator procedures; it attributes the multicenter analysis reporting 38.1% success to protocol deviation, and another trial’s results to an insufficient learning curve [3].

Each criticism may be correct, and at least one is demonstrably substantive: the multicenter analysis required only three consecutive or five total treatments for inclusion [30], well below the specified ten-session course, so some patients received a sub-therapeutic dose by the therapy’s own standard—a fair criticism, identifiable from the methods without privileged knowledge.

The difficulty is structural. A framework in which fidelity is assessed after the result is known, by the party whose theory the result bears on, and in which no prospective operationalized fidelity criterion exists against which a trial could be judged compliant before it reports, is a framework in which no trial can disconfirm the hypothesis. Every positive result confirms the therapy; every negative result impugns the delivery. This is not a criticism of good faith; the concerns are substantive and have been consistent for two decades. It is a criticism of the field’s failure to convert them into instruments.

The same asymmetry can enter interpretive reviews, including this one. Some negative trials may reflect a genuine absence of benefit, or a benefit too small to be clinically meaningful, rather than implementation failure, and that possibility should be held open with the same weight as the implementation explanations above. We have tried to apply equal scrutiny to positive trials—the allocation and ascertainment features of the trial reporting the largest effect [4], and the blinding and attrition features of the post-burn imaging trial [18] are examples—but readers should judge whether that scrutiny has been symmetrical.

The remedy is within reach. Fidelity criteria that are already stated qualitatively—intra-session elimination of pain, electrode placement outside the painful field, and repositioning when abnormal sensation persists—should be specified prospectively, measured session by session, and reported as a fidelity outcome alongside efficacy. A trial meeting pre-registered fidelity criteria and still reporting no benefit would be informative in a way no current trial is. An instrument already exists—the developer describes a free data-management application designed to document each treatment and reduce operator-dependent bias [3]—and we could identify no published trial reporting its use. The tools for falsifiability exist; what is missing is the requirement to use them.

### 7.7. Steps Toward Standardization

Table 2 sets out a proposed preliminary 22-item reporting set across six domains: device identity, treatment topology, dose and titration, operator qualification, concomitant therapy, and outcome architecture. It is intended to complement CONSORT and TIDieR rather than replace them and is designed so that a reader can reconstruct the intervention from the report.

We are explicit about what this is not. Established reporting guidelines emerge from structured consensus—candidate-item review, multi-round Delphi, a consensus meeting—and the ST-MRS has undergone none. It is a single-group proposal by the five authors of this review, two of whom hold office in a society with a declared interest in the modality, and should be read as an agenda item for a consensus process rather than its output. Independent external evaluation, by investigators with no affiliation to the modality, would be required before it could serve as a reporting standard.

Item selection and priority: Items were selected using a single rule: each corresponds to a deficiency documented in the primary record reviewed above, and none were included on theoretical grounds alone. We did not weight items formally, but they differ in function. Items 3, 6, 10, 12, 18, and 22 bear most directly on internal validity, because their omission prevents a reader from determining whether the intervention delivered was the intervention claimed, whether a candidate effect modifier was balanced, or whether blinding held; the remaining items bear principally on reproducibility. A consensus process should test both the item list and this prioritization.

Relationship to existing guidance: Many items overlap with existing reporting guidelines, and the ST-specific contribution is narrower than the table’s length suggests. TIDieR already requires reporting of materials (TIDieR item 3), who provided the intervention and their training (item 5), when and how much (item 8), tailoring (item 9), and planned and actual fidelity (items 11–12); these correspond to ST-MRS items 1, 13–14, 9, 8, 10–11, and 12, respectively. CONSORT 2010 requires pre-specified outcomes and their timing (item 6a), as well as a description of blinding and the similarity of interventions (items 11a–b), corresponding to ST-MRS items 20 and, in part, 22; it does not require formal assessment of blinding integrity, which item 22 adds. SPIRIT requires the corresponding elements at the protocol stage. Elements not required by any of these guidelines, and which arise from the specific characteristics of ST, are the regulatory designation of the device (items 2–3), the electrode-topology rule and its bracketing requirement (items 5–7), the intra-session titration endpoint (item 10, which TIDieR’s tailoring item accommodates but does not specify), inter-operator agreement on placement (item 16), separate reporting of sodium-channel-blocking anticonvulsants (item 18), and endpoint timing justified against the expected effect trajectory (item 20). Where an item is already covered, the ST-MRS specifies what an ST report should contain under that heading rather than duplicating it.

We are not alone in this diagnosis: a 2025 review by authors with no society affiliation names the lack of standardization as a principal challenge and calls for studies comparing session lengths and counts [50]; the developer’s reply contests aspects of that appraisal [51]. Standardization infrastructure also partly exists—a structured three-day training course on a train-the-trainer model, restriction of use to physicians or supervised qualified personnel, specific electrode requirements, and the data-management application noted above [3]. The deficiency is therefore not an absence of standards but an absence of any requirement that trials must state whether they met them.

Table 3 presents a template for a phenotype-stratified dismantling trial addressing the sham paradox, specifying operator competency, fixing device identity, stratifying on concomitant medication, and adopting a day 28–42 primary endpoint. Given the demonstrated differential response by phenotype [20], randomization should be stratified based on baseline Neuropathic Pain Symptom Inventory clusters, or, at minimum, a powered paroxysmal-versus-persistent subgroup analysis; pre-specified aggregate analyses averaging across phenotypes with different response profiles will fail to replicate.

Several elements of Table 3 require development before it can serve as a protocol, and we present it as a conceptual research framework. Perceptual matching between Arms A and B should be verified rather than assumed: participants would rate the intensity, quality, and location of sensation on standardized scales after each session, with the arms compared against a pre-specified equivalence margin in a pilot phase before the main trial. Fidelity would be monitored by session-level checklists recording electrode placement (photographed), final intensity, and achievement of the titration endpoint, audited by an independent reviewer on a random sample of sessions. Because the operator cannot be blinded to the arms, the operator–participant interaction should be scripted, operators should not collect outcomes, and assessors and analysts should be separated from delivery. A treatment-by-phenotype interaction requires a substantially larger sample than the main effect—approximately four times as large to detect an interaction of the same magnitude—so the comparison of Arms A and B should be the single primary hypothesis, with the phenotype interaction and Arm C tested hierarchically to control for multiplicity. The minimal clinically important between-group difference should be pre-specified and justified; it is expected to be smaller than the within-patient thresholds of approximately 2 points or the 30% commonly used to define response, and the trial should be powered against the former. Changes in concomitant analgesia should be governed by protocolized rescue and tapering rules and analyzed both as a secondary outcome and as a time-varying covariate in sensitivity analyses.

Explanatory trials cannot answer what happens when ST is delivered at scale by operators of varying experience; Table 4 specifies a minimum prospective registry dataset with mandatory device identity, operator credential, protocol adherence, and 90-day follow-up.

### 7.8. The Korean Case: Administrative Diffusion Without Standardization

In the Republic of Korea, ST is delivered as a non-covered service, and providers report charges to the Health Insurance Review and Assessment Service (HIRA), which publishes institution-level price information by administrative region [52]. These public records are useful for examining how the intervention is classified administratively, but they are not a census of patients treated or of institutions confirmed to deliver Scrambler Therapy^®^.

Two features of the classification matter. The disclosed item is designated non-invasive, painless signal therapy, fee code MZ012—a technique name carrying no manufacturer, model, or proprietary designation. The governing regulatory notice is not device-agnostic: its official procedure definition specifies delivery by the Pain Scrambler MC-5A [13]. The current public disclosure record, however, does not include a device field [52]. Consequently, the model specified at the rule level cannot be verified from the price-disclosure record. The item is filed under physical therapy, in the physical-medicine fee chapter, rather than in a neuromodulation or interventional pain category.

The national trajectory shows why this distinction matters. The technique was recognized as safe and effective in November 2012, gazetted under its Korean technique name in February 2013, and listed as a non-covered service from June 2014. At the 2021 national reassessment, 189 institutions disclosed a price, ranging from KRW 30,000 to 150,000 per session, while certainty of efficacy was rated as very low, and the recommendation was conditional [13]. The agency specifically requested blinded comparative studies, long-term outcome data, and cost-effectiveness analysis. To characterize subsequent administrative diffusion, we queried the HIRA non-covered treatment cost disclosure system region by region on 18 August 2026 and summed institutions reporting a price under the same administrative item [52]. This returned 871 institutions, approximately 4.6 times the 2021 count. Across the same five-year interval, the evidence requirements identified by NECA had not been met in the published literature reviewed here. Administrative diffusion accelerated; the evidentiary gaps remained.

Three observations follow.

First, the public classification cannot verify device identity. Because the disclosure record carries no device field, analyses based on this administrative item cannot determine whether every record corresponds to the device specified in the governing notice [13,52]. This creates a structural risk of misclassification whenever administrative records are interpreted as evidence about a specific device-defined intervention.

Second, administrative category counts cannot be equated with the confirmed adoption of a specific device. The disclosure record identifies institutions reporting a price under the technique name, not the device deployed, protocol fidelity, patient volume, indication, or treatment outcome. The figure of 871 institutions therefore quantifies the national diffusion of the administrative category, not confirmed uptake of Scrambler Therapy^®^ or any particular device.

Third, price disclosure is consistent with—but does not establish—delivery heterogeneity. In the HIRA snapshot from 18 August 2026, reported charges ranged from KRW 1000 to 500,000 per session, compared with KRW 30,000 to 150,000 in 2021 [13,52]. The lower extreme may reflect placeholder reporting, and the system publishes no distribution summary, so the range should not be interpreted as a treatment-effect or quality measure. Nevertheless, a five-hundred-fold span under one per-session administrative item is consistent with substantial variation in what is being reported under that label, while the public record still lacks device, session duration, channel count, and operator training fields.

What these public records cannot provide is a denominator for confirmed Scrambler Therapy^®^ use. Because the service is non-covered, it does not generate a standard reimbursed claim that identifies patients, indications, or devices. Korea can therefore disclose institutional prices under the administrative category without establishing how many patients receive confirmed Scrambler Therapy^®^, for what indications, with what device, or with what protocol fidelity. Real-world evidence for the modality must therefore be built deliberately through a registry such as that specified in Table 4.

That conclusion assigns a specific and non-decorative role to national professional societies: a society maintaining a curriculum, a certification pathway, and a published protocol can provide one practical infrastructure through which a non-covered, operator- and device-dependent modality may generate interpretable real-world data. The authors’ society affiliation is disclosed below, and readers should weigh this argument accordingly.

## 8. Future Directions

Six priorities are listed as follows, in order of the expected information yield per unit cost:

1. A dismantling trial comparing full-protocol ST against a perceptually identical fixed-waveform or in-field-electrode control, in one well-characterized syndrome, powered for a pre-specified endpoint (we propose day 28–42). This is the clearest direct test of whether the proposed active principle contributes specifically to the observed effect.
2. Full publication or formal retrieval of the 2013 active-sham trial [48], or its replication at adequate size. A field cannot leave its most design-relevant result in a conference supplement.
3. A phenotype-stratified confirmatory trial in postherpetic neuralgia or painful diabetic polyneuropathy, stratified on phenotype cluster and baseline sodium-channel-blocker use, with blinding integrity reported.
4. A device-stratified re-analysis of the existing meta-analytic dataset, separating confirmed ST-device studies from label-only studies, and re-examining whether trials classified as sham-controlled delivered inert shams.
5. A prospective multicenter registry with the Table 4 dataset, and an independent synthesis commissioned from investigators with no primary-trial involvement.
6. Mechanistic imaging and neurophysiology extending the cerebral blood volume findings [18] with pre-specified network hypotheses, and quantitative sensory testing relating response to sensory phenotype.

## 9. Conclusions

Scrambler therapy has a coherent and distinctive proposed mechanistic rationale, a reproducible direction of effect across two decades and multiple independent groups, evidence of post-treatment persistence that is difficult to explain by gating and compatible with, though not proof of, its proposed mechanism, and a reported harm profile that compares favorably with the pharmacological alternatives in refractory neuropathic pain, although, as set out above, that profile rests on largely passive ascertainment and is better read as a lower bound. It also has an evidence base that guideline bodies have correctly declined to endorse, because that base is composed of studies that cannot be meaningfully combined.

The remedy is not simply larger trials. It is trials that specify their device, report their electrode topology, document their titration endpoint, state their operators’ qualifications, control for concomitant sodium-channel blockade, resolve or explicitly measure their blinding, verify that their sham is inert, and measure their outcome when the proposed mechanism predicts the effect will be present. We do not claim to have demonstrated this; a narrative review cannot. We claim only that the six problems set out here are documented in the primary record, could plausibly account for much of the observed non-convergence, and are addressable. On this basis, we argue that standardization is a major rate-limiting step, and that until it is addressed, additional trials risk adding volume to the literature without adding information to it.

Standardization is a means of making results interpretable, not a means of making them favorable. A rigorously standardized, adequately powered, independently conducted trial might show that scrambler therapy confers little or no clinically meaningful benefit over an appropriate control, and some negative trials already published may prove to have been correct. Either outcome would serve patients and payers better than the present state of the literature.

## Figures and Tables

**Figure 1:**
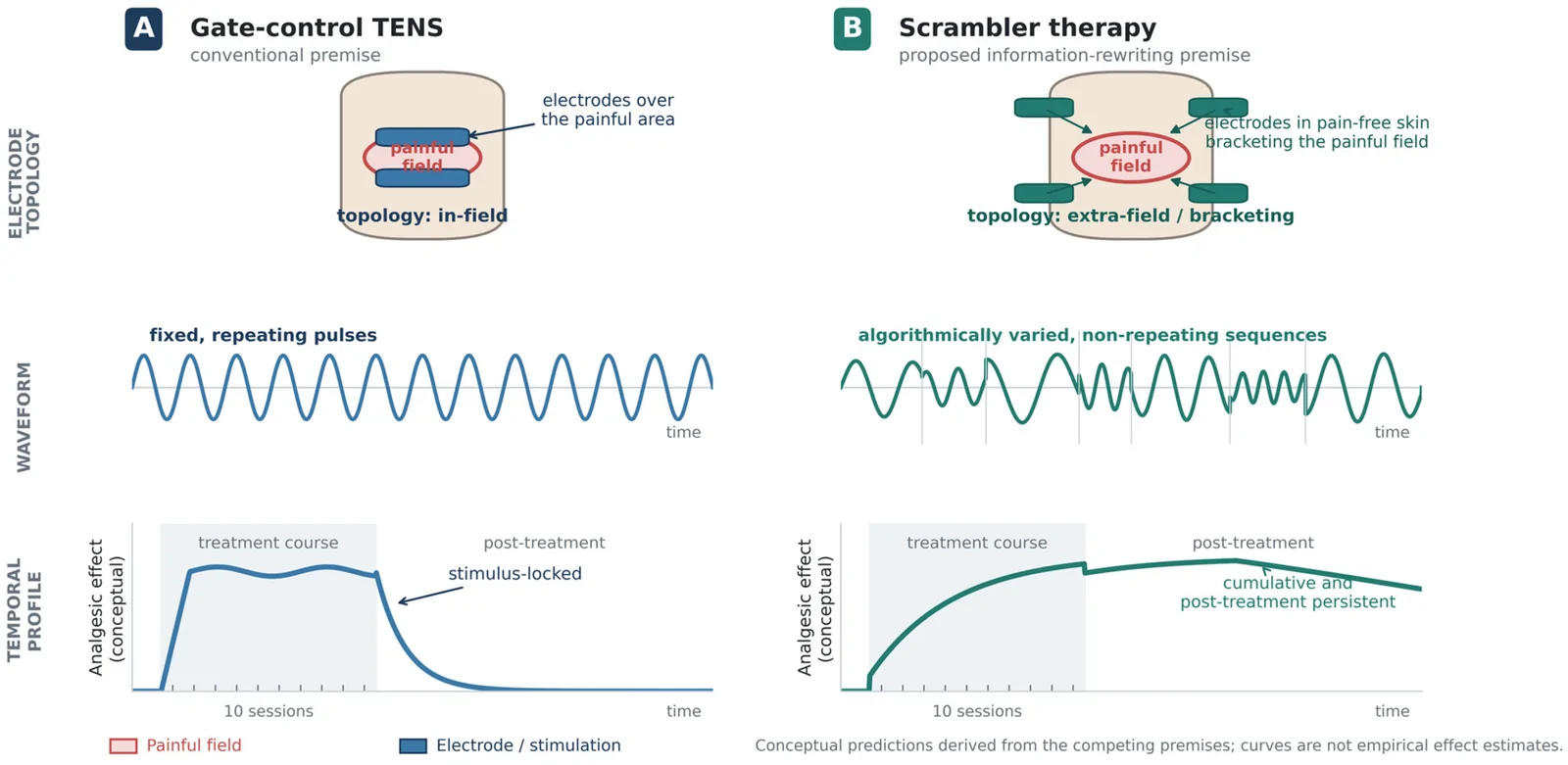
Conceptual contrast between (**A**) gate-control-based TENS and (**B**) the proposed information-rewriting premise of scrambler therapy, including electrode topology relative to the painful field, waveform structure, and the predicted temporal profile of analgesia (stimulus-locked versus cumulative and post-treatment-persistent). The temporal curves are conceptual predictions and are not empirical effect estimates.

**Figure 2:**
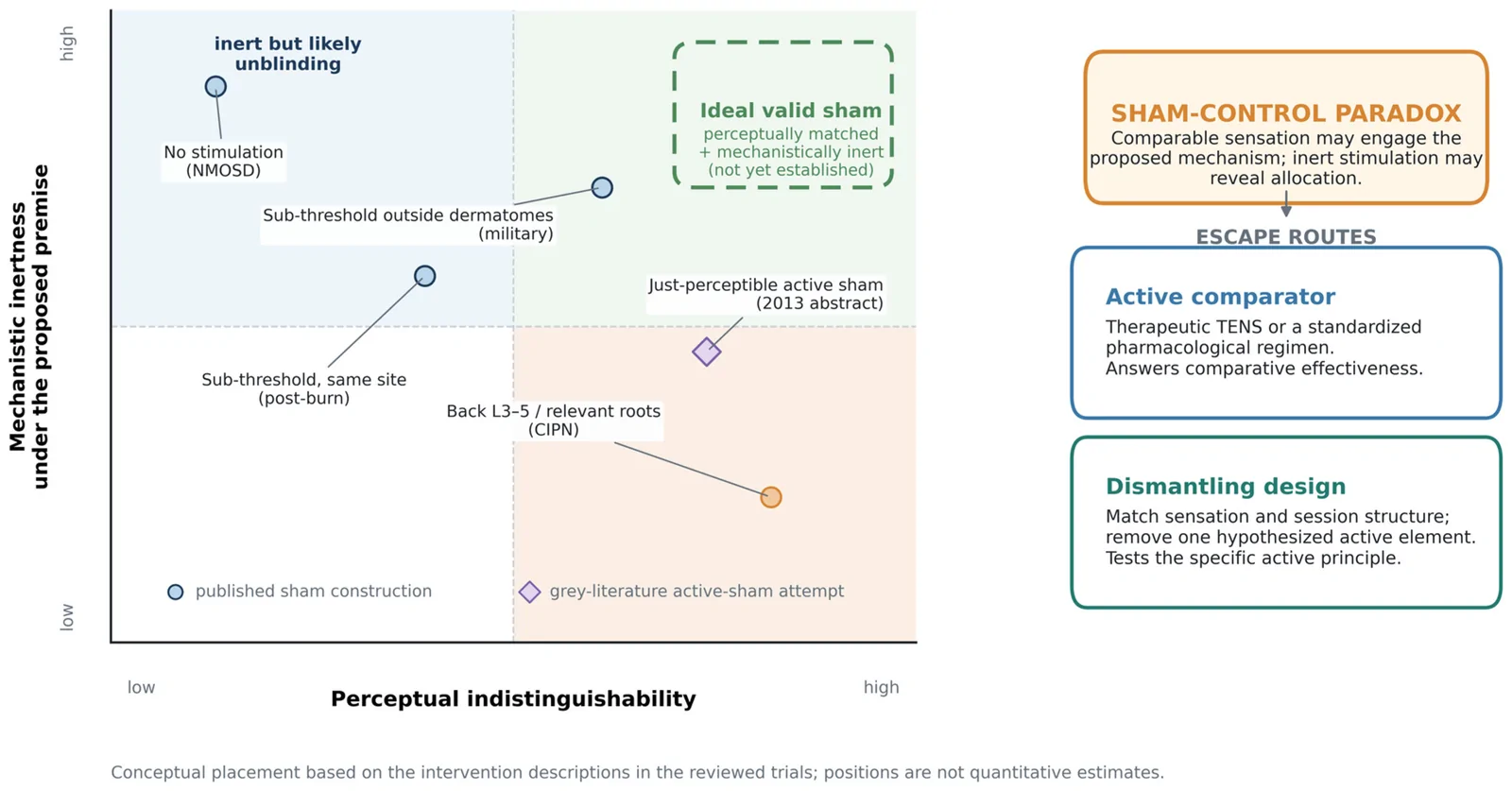
The sham paradox in scrambler therapy trial design: the mutual exclusivity of perceptual indistinguishability and mechanistic inertness under current assumptions. Published and gray-literature sham constructions are positioned conceptually within the matrix, with active-comparator and dismantling designs shown as escape routes. Placements are based on intervention descriptions and are not quantitative estimates.

**Table 1:** Principal controlled and prospective studies on scrambler therapy.

Study	Design	Population (*n*)	Comparator	Principal Finding
Marineo et al., 2012 [4]	Controlled trial; alternate allocation (allocation not concealed); outcomes collected by the unblinded interventionists; anticonvulsant users excluded	Mixed drug-resistant neuropathic pain (52)	Guideline-based pharmacotherapy	VAS 8.0 → 0.78 (91%) vs. 8.1 → 5.84 (28%) at 1 month, sustained at 3 months; reduced analgesic use; allodynia differed at 1 and 2 months but not at 3 months
Smith et al., 2010 [21]	Pilot, single-arm design	CIPN	—	Substantial symptomatic improvement
Coyne et al., 2013 [22]	Prospective, open	Cancer pain and chronic CIPN	—	Consistent symptomatic benefit
Starkweather et al., 2015 [23]	Double-blind RCT	Chronic low back pain	Sham	Reduced pain intensity; differential gene expression
Ricci et al., 2019 [24]	Large series, 15-year duration	Chronic pain (219)	—	Durable reduction maintained through follow-up
Mealy et al., 2020 [17]	RCT, single-blind; single trained technician delivered all sessions; GEOMC MC-5A	Neuromyelitis optica spectrum disorder (22; 11 per arm)	Sham: device powered on, no stimulation emitted	Primary outcome feasibility/acceptability. Median NRS 5.0 → 1.5 vs. no significant change in sham; anxiety and depression improved; sustained at 30 days but not at 60 days
Loprinzi et al., 2020 [25]	Randomized phase II pilot	CIPN	TENS	Equivocal separation
Smith et al., 2020 [26]	Pilot RCT, sham-controlled (30 min sessions)	Chronic CIPN (35; pain data for 33)	Sham = treatment on the back at L3–5, at the segmental entry of the relevant nerve roots	No difference at day 10, 28, 60, or 90 in average pain, BPI, or EORTC CIPN-20. Responses during active treatment largely dissipated by day 30. All active-arm patients wished to continue
Min et al., 2021 [20]	Prospective, open-label, single-arm (GEOMC NPC3 device; manufacturer-funded)	Chronic drug-resistant neuropathic pain, mixed etiology (27)	—	NRS −15% (95% CI −28 to −2.4); differential response by NPSI phenotype cluster (*p* = 0.003)
Lee et al., 2022 [18]	RCT, participant- and assessor-blind (MC-5A Calmare; 45 min sessions)	Chronic post-burn neuropathic pain (43 randomized; 37 analyzed)	Sub-threshold sham, same site	VAS 6 → 3 vs. sham 7 → 6 (between-group *p* < 0.001); sham also improved from baseline; CBV decreased in frontal regions, increased in pre-/postcentral gyri. Six active-arm patients lost to follow-up owing to improvement
Nayback-Beebe et al., 2020 [27]	Double-blind RCT (30 min sessions)	Chronic traumatic neuropathic pain, military service members (57 randomized; 47 analyzed)	Sham: sub-threshold intensity, leads outside affected dermatomes	No between-group difference in pain, analgesic use, or HrQoL. Both arms improved (4.3 → 2.5 vs. 4.8 → 3.2). In total, 90% of blinded participants rated the treatment a partial or complete success, with no difference between arms
Stowell-Campos et al., 2024 [28]	RCT, patient- and assessor-blind; five 40 min sessions	Post-stroke contralesional pain (20)	Sham	Mean pain change −3.73 vs. −0.94 post-intervention, −2.57 vs. −0.25 at 4 weeks (*p* = 0.012, 0.004); >50% reduction: 70% vs. 10% immediately (*p* = 0.006), 30% vs. 10% at 4 weeks (ns)
Yoo et al., 2023 [29]	Single-arm prospective pilot; ten 45 min sessions	Painful diabetic polyneuropathy (9)	—	VAS improved at sessions 8–10 and at 1 month; all other outcomes and time points to 6 months not significant
Moon et al., 2015 [30]	Multicenter retrospective analysis, 3 centers; inclusion required only ≥3 consecutive or ≥5 total sessions	Mixed chronic pain (147)	—	Success rate of 38.1%. Independent predictors of success: neuropathic pain (OR 24.78), mixed pain (OR 10.52), and treatment site (OR 6.87 and 12.29). Membrane stabilizer use not predictive; antidepressant use borderline for failure
Childs et al., 2021 [31]	Randomized crossover analysis	CIPN	TENS	Crossover phase did not resolve the ambiguity of the primary comparison
Kashyap et al., 2020 [32]	RCT, open	Head, neck, and thoracic cancer pain (80)	Pharmacological standard of care; no TENS or sham	Greater pain reduction and reduced opioid dose with ST; follow-up 1 week beyond final session
Kashyap et al., 2022 [33]	RCT	Cancer pain, palliative setting	—	Improvement in quality of life
Chung et al., 2024 [34]	Pilot study	CIPN	—	Improvement in worst pain, numbness, and tingling sustained at 6 months

BPI, Brief Pain Inventory; CBV, cerebral blood volume; CI, confidence interval; CIPN, chemotherapy-induced peripheral neuropathy; EORTC, European Organisation for Research and Treatment of Cancer; HrQoL, health-related quality of life; NPSI, Neuropathic Pain Symptom Inventory; NRS, numerical rating scale; OR, odds ratio; RCT, randomized controlled trial; TENS, transcutaneous electrical nerve stimulation; VAS, visual analog scale.

**Table 2:** Scrambler Therapy Minimum Reporting Set (ST-MRS): a proposed preliminary framework, not a validated reporting standard.

#	Domain	Item
1	Device identity	Manufacturer, model, and software/firmware version
2	Device identity	Regulatory clearance identifier and jurisdiction
3	Device identity	Statement of whether the device is Scrambler Therapy^®^-designated or an alternative electroanalgesic device
4	Device identity	Number of channels available and number used
5	Treatment topology	Anatomical rule used for electrode siting (dermatomal, peripheral nerve, or other), stated explicitly
6	Treatment topology	Confirmation that electrodes were placed in pain-free skin bracketing the painful field, with any exceptions
7	Treatment topology	Diagram or photograph of representative electrode placement
8	Treatment topology	Protocol for repositioning when the perceptual endpoint is not achieved
9	Dose and titration	Session duration, number of sessions, and inter-session interval
10	Dose and titration	Defined endpoint for intensity titration and how it was verified, including whether intra-session pain elimination (to 0 or to <2/10) was achieved and recorded for each session
11	Dose and titration	Mean and range of final intensity settings achieved
12	Dose and titration	Number of sessions completed per patient; definition and rate of protocol completion
13	Operator	Professional discipline of operators
14	Operator	Training received: hours, supervised cases, and whether competency was formally assessed
15	Operator	Number of operators and cases per operator
16	Operator	Inter-operator agreement on electrode placement, where assessed
17	Concomitant therapy	Baseline analgesic regimen by class and dose
18	Concomitant therapy	Sodium-channel-blocking anticonvulsant use at baseline, reported separately
19	Concomitant therapy	Whether concomitant therapy was held stable, tapered by protocol, or unrestricted
20	Outcome architecture	Primary endpoint timing, with justification relative to expected effect trajectory
21	Outcome architecture	Follow-up to a minimum of 90 days; responder analysis at ≥30% and ≥50%
22	Outcome architecture	Blinding-integrity assessment: patient and operator allocation guesses, reported

**Table 3:** Template design for a phenotype-stratified dismantling trial.

Element	Specification
Population	Single syndrome (e.g., postherpetic neuralgia), pain ≥ 4/10 for ≥3 months, refractory to ≥1 guideline-concordant pharmacological trial
Stratification	(a) NPSI cluster: paroxysmal-predominant vs. persistent-predominant; (b) baseline sodium-channel-blocker use: yes/no
Arm A	Full-protocol ST: algorithmically varying sequences, multi-channel, electrodes in pain-free skin bracketing the painful field, operator titration to defined perceptual endpoint
Arm B (dismantling control)	Perceptually matched fixed repeating waveform at identical amplitude, identical electrode topology, and session structure
Arm C (optional)	Full waveform algorithm with electrodes placed within the painful field
Blinding	Patient- and assessor-blind; operator necessarily unblinded to arm assignment but not to hypothesis ranking; allocation guesses collected at study end from patients, operators, and assessors
Dose	Ten consecutive daily sessions, 35–45 min, weekdays over two weeks
Primary endpoint	Change in average pain NRS from baseline to day 35 (window: 28–42) (proposed; see text)
Key secondary	End-of-course NRS (day 10); NRS at day 90; BPI interference; analgesic consumption with protocolized tapering rules; ≥30% and ≥50% responder rates; blinding-integrity index
Analysis	Mixed-effects model with a pre-specified time × arm × phenotype-cluster interaction

**Table 4:** Minimum prospective registry dataset.

Domain	Fields
Patient	Age, sex, pain duration, diagnosis (ICD code), pain distribution, prior interventions
Phenotype	Baseline NPSI, NRS, BPI interference; presence of allodynia
Device	Manufacturer, model, clearance identifier
Delivery	Sessions delivered and completed; channels used; electrode region; final intensity; operator identifier and credential level
Concomitant	Analgesic classes and doses at baseline, end of course, and 90 days; sodium-channel blockers flagged
Outcome	NRS and BPI interference at day 10, day 35, and day 90; adverse events; discontinuation with reason
Linkage	Where permitted, subsequent analgesic prescribing, procedural escalation, and healthcare utilization

BPI, Brief Pain Inventory; ICD, International Classification of Diseases; NPSI, Neuropathic Pain Symptom Inventory; NRS, numerical rating scale.

## Data Availability

No patient-level or experimental data were generated. The literature and national health-technology reassessment information are available in the cited sources. The administrative observations in the section on the Korean case were derived from the publicly accessible HIRA non-covered treatment cost disclosure system [52], queried region by region on 18 August 2026 because the system does not provide a national total. The reported 871-institution count and price range are reproducible by repeating those regional queries as described in the text.
